# Population-based study of diagnostic assays for Borrelia infection: comparison of purified flagella antigen assay (Ideia™, Dako Cytomation) and recombinant antigen assay (Liaison^®^, DiaSorin)

**DOI:** 10.1186/1472-6890-8-4

**Published:** 2008-04-18

**Authors:** Eskild Petersen, Martin Tolstrup, Francesco Capuano, Svend Ellermann-Eriksen

**Affiliations:** 1 Department of Infectious Diseases, Aarhus University Hospital Skejby, Aarhus, Denmark; 2 Department of Clinical Microbiology, Aarhus University Hospital Skejby, Aarhus, Denmark; 3 DiaSorin S.p.a., Saluggia, Italy

## Abstract

**Background:**

Testing for *Borrelia*-specific IgM and IgG-antibodies are often performed on a variety of poorly defined symptoms, and isolated IgM results are a frequent finding, which results in diagnostic uncertainty and further testing. We wanted to test the hypothesis that Borrelia-specific assays using recombinant antigens perform differently from assays based on purified flagella antigen.

**Methods:**

We compared the use of recombinant antigens (LIAISON^® ^DiaSorin, Saluggia, Italy) and purified flagella antigen (IDEIA™ Borrelia, DakoCytomation, Glostrup, Denmark) in the assay for *Borrelia*-specific IgM and IgG-antibodies. The assays were tested on an unselected population of serum samples submitted from general practice. A total of 357 consecutive samples for analysis of *Borrelia *IgM and IgG antibodies. Furthermore, we analysed 540 samples for *Borrelia*-specific IgM or IgG antibodies first by the IDEIA™ and, if they were positive, the samples were further analysed using the LIAISON^® ^assay. To verify the correctness of the patient's serological status, discrepant samples were analysed by line blots (EcoLine, Virotech).

**Results:**

In the consecutive series of 357 samples, the IgM assays detected 308 negative and 3 positive samples with concordant results. Compared with the line blot, the IDEIA™ system produced 21 false-positive IgM results, whereas the LIAISON^® ^system produced only one false-positive IgM result. The IgG assays showed 1 positive and 328 negative concordant results. The LIAISON^® ^system produced 9 true IgG-positive samples that were not detected by the IDEIA™ system, but the former produced 4 positive IgG results that were negative by line blot.

**Conclusion:**

Diagnostic assays based on flagella antigen seem to show more false-positive IgM and false-negative IgG results than assays based on recombinant antigens. The latter may reduce the number of presumably false-positive IgM results and identify more IgG-positive subjects, but this system also produces more false-positive IgG results.

## Background

Infection with *Borrelia *spp. is the most common vector borne infection in Europe with an estimated more than 60,000 symptomatic cases annually and a reported incidence from Germany of about 1 per 1,000 population [1]. Infection may result in a variety on clinical symptoms, including neuroborreliosis with cranial nerve paresis and radicular pain. *Borrelia *infections in humans are caused by *B. afzelli*, *B. garinii *and *B. burgdorferi *sensu stricto. A study from Germany found that 65% of patients were infected with *B. garinii*, 24% with *B. afzelii *and 11% with *B. burgdorferi *sensu stricto [2]. Clinical borrelia infection is divided in three stages, 1) erythema migrans; 2) multiple erythema migrans, lymphadenosis benigna cutis, neuroborreliosis and myocarditis; and 3) arthritis, acrodermatitis chronica athrophicans and chronic neuroborreliosis [3].

The diagnosis of neuroborreliosis is difficult. It relies primarily on clinical signs, demonstration of intrathecal antibody synthesis and pleocytosis in the spinal fluid [4]. Antibody based assays suffers from lack of standardization [5], and borrelia infection is therefore overdiagnosed based on vague criteria and equivocal serological results especially IgM positivity without specific IgG-antibodies [6]. Nucleic acid based molecular diagnosis has an even lower sensitive [7].

*Borrelia *spp. is believed to express different antigens in different hosts [8], gene expression is reduced as the *Borrelia *adapt to the host [9,10] and consequently serological diagnosis is difficult and unpredictable in the early stages of the infection. This partly explains why common consensus on diagnostic criteria is lacking [11]. The flagella contain the immunodominant 41 kDa, flagellin antigen, and several outer surface proteins, Osp's. The use of an enzyme-immunoassay followed by a Western blot or dot blot assay has not improved diagnostic accuracy [12]. However, Western blotting with a mixture of *B. burgdorferi *sensu stricto and *B. afzelii *antigens have been found to improve diagnosis in up to 70% of sera with borderline results [13], but Western blot or immunoblots using recombinant antigens can not be regarded as a golden reference standard [14]. Antibody assays have not been useful in the diagnosis of erythema migrans. Patients with systemic *Borrelia *infections show IgG-antibodies after up to eight weeks after infection [15]. Persistent IgM-antibodies with without development of *Borrelia*-specific IgG-antibodies is usually regarded as false-positive reactions [15].

The two diagnostic assays have previously been evaluated on patients with well defined clinical borrelia infection in different stages. In patients with erythema migrans, the flagella assay were found to have a sensitivity of 44.8% in the IgM assay and 35.5% in the IgG assay [14], while the recombinant antigen showed a sensitivity of 46.9% in the IgM assay and 66.3% in the IgG assay [16].

In patients with neuroborreliosis the flagella assay displayed a sensitivity of 67.9% for the IgM assay an 76.8% for the IgG assay [15], whereas the recombinant antigen assay was found to have a sensitivity of 36.8% for the IgM assay an 90.8% for the IgG assay [16].

The aim of the present study was to evaluate chemiluminescence immuno assays based on recombinant antigens (LIAISON^®^, DiaSorin, Salugia, Italy) and assays based on purified flagella antigen (IDEIA™, Dako Cytomation, Glostrup, Denmark). This comparison was performed on a population based panel of samples submitted from general practice for analysis for *Borrelia *IgM- and IgG-antibodies. In order to compare the two assays, samples with discordant outcome were analysed by a Lineblot (EcoLine, Virotech) to more correctly ascertain the serological status of the patient.

## Methods

### Samples

Eight hundred and ninety-seven serum samples submitted primarily from general practitioners for analysis of *Borrelia*-specific IgM and IgG antibodies were included. We included 357 consecutive samples which were all tested by the recombinant antigen chemiluminescence immuno assay (LIAISON^®^) and the purified flagella antigen assay (IDEIA™ *Borrelia*) independent of the results from the other test systems, and samples were recruited no matter whether submitted from general practice or from hospitalized patients. Samples with equivocal results were included. Patients have not been selected based on sex or age. All patients were permanent residents in Denmark. Patients occurred only once in each dataset. These consecutive samples were collected from December 2003 to May 2004 and the selected samples from the end of January to the end of April 2004. The five hundred and forty samples collected from January to December 2003 were tested first by purified flagella antigen test (IDEIA™ *Borrelia*, DakoCytomation, Glostrup, Denmark) for *Borrelia*-specific IgM and IgG antibodies and samples with a positive IgM or IgG result from this analysis were further analysed in the recombinant antigen test (LIAISON^®^, DiaSorin, Saluggia, Italy). Samples showing discrepancy in either IgM or IgG results from these tests were analysed by line blot (EcoLine, Virotech).

All samples were anonymised before entered into the study. Studies on anonymised samples do not require approval by a scientific ethical committee according to Danish law.

### Assays

#### Purified flagella antigen enzymeimmuno assay (IDEIA™ *Borrelia burgdorferi*, DakoCytomation, Glostrup, Denmark)

The samples were tested by the standard assay applied by the Department of Clinical Microbiology, using the *Borrelia burgdorferi *purified flagella antigen, IgM μ-capture assay and IgG sandwich ELISA assay from DakoCytomation according to the instructions provided by the manufacturer as described by Hansen (1994) [14]. The diagnostic sensitivity for the assay is in patients with eythema migrans 44.8% for the IgM assay and 35.5% for the IgG assay [14], in patients with neuroborreliosis 67.9% for the IgM assay and 76.8% for the IgG assay [14] and in patients with acrodermatitis 12% for the IgM assay and 100% for the IgG assay [14]. We have not been able to find data on specificity for this assay.

A positive test was defined according to the manufacturer's recommendations. As proposed by DAKO Cytomation, readings +/- 10% of the cut off value for IgM and +/- 20% of the cut off value for IgG were considered as equivocal. Values higher than these were considered as positive.

#### Recombinant antigen enzymeimmuno assay (LIAISON^®^, DiaSorin, Saluggia, Italy)

The samples were tested by recombinant *Borrelia *IgM and IgG assays. A positive test is defined according to the manufacturer's recommendation. Samples with *Borrelia burgdorferi *IgG concentrations equal or above 15 AU/ml were graded positive. Samples with *Borrelia burgdorferi *IgM concentrations equal or above an index value of 1.1 were graded positive. The assay is an indirect chemiluminescence immunoassay based on recombinant antigens produced in *E. coli*. LIAISON^® ^*Borrelia *IgM utilize a solid phase coated with recombinant Osp C, *Borrelia afzelii*, strain pKo. OpsC is immunodominant for the IgM response. LIAISON^® ^*Borrelia *IgG make use of recombinant VlsE, *B. garinii*, strain pBi, an outer surface lipoprotein [17]. In patients with erythema migrans the IgM assay has a sensitivity of 46.9% and the IgG assay 66.3% [15], in patients with neuroborreliosis 36.8% for the IgM assay an 90.8% for the IgG assay [15]. The diagnostic specificity of a negative IgM test is 99% and of a negative IgG test 98%[15]. The code number of the Borrelia IgM version used in the study is 310890 and the code for Borrelia IgG is 310880.

#### Borrelia EcoLine^®^, *Borrelia burgdorferi*, (Virotech, Rüselheim, Germany)

The test principle is a series of dot blots with recombinant and purified native *Borrelia *antigens blotted onto a nitrocellulose strip. The IgM-test includes OspC, VlsE, BmpA and an Epstein-Barr virus antigen. The IgG-test includes recombinant VlsE, BmpA, p83, BBA36, BBO323, Crasp3 and pG, but it is not stated which Borrelia subspecies the different antigens comes from. The tests were interpreted as recommended by the manufacturer.

### Statistics

The two assays were compared using Bowker's test for symmetry using SAS version 9.1 [18]. The test makes no assumption on true positive or negative results of the two tests but only test symmetry in the two by two tables.

## Results

### Consecutive samples

The concordance between the recombinant antigen assay (LIAISON^®^) and the purified flagella antigen assay (IDEIA™) for *Borrelia*-specific IgM- and IgG-antibodies from the series of consecutive samples is summarized in table 1 and 2. Out of the 357 consecutive samples collected from December 2003 to May 2004, 308 were negative and 3 were positive in both of the IgM assays (Table 1. Bowker's test statistic 25.7; p < 0.0001). In the IgG-assays, one tested positive for *Borrelia*-specific IgG-antibodies in both the recombinant antigen test (LIAISON^®^) and the purified flagella antigen test (IDEIA™) and 328 tested negative by both assays (Table 2. Bowker's test statistic 25.3; p < 0.0001).

**Table 1:** *Borrelia *specific IgM comparing recombinant antigen assay (LIAISON^®^) and purified flagella assay (IDEIA™). Consecutive, unselected samples.

		Purified flagella *Borrelia *IgM (IDEIA™)			
			
		POS	EQV	NEG	
Recombinant antigen *Borrelia *IgM (Liaison^®^)	POS	3	1	6	10
	EQV	0	0	0	0
	NEG	33	6	308	347
		36	7	314	357

Bowker's test statistic 25.7; p < 0.0001

**Table 2:** *Borrelia *specific IgG comparing recombinant antigen assay (LIAISON^®^) and purified flagella assay (IDEIA™). Consecutive, unselected samples.

		Purified flagella *Borrelia *IgG (IDEIA™)			
			
		POS	EQV	NEG	
Recombinant antigen *Borrelia *IgG (LIAISON^®^)	POS	1	2	17	20
	EQV	1	0	8	9
	NEG	0	0	328	328
		2	2	353	357

Bowker's test statistic 25.3; p < 0.0001

Thirty-three of the consecutive samples were IgM positive in the purified flagella antigen test (IDEIA™) assay but negative by the recombinant antigen IgM test (LIAISON^®^). These samples were further tested by the Borrelia EcoLine^® ^line blot and 63.6% (21/33) were negative, 12.1% (4/33) were Epstein Barr Virus positive and potentially cross reactive, 9.1% (3/33) were equivocal and 5/33 (15.2%) were positive (Table 3). Six samples were IgM negative in the purified flagella antigen test (IDEIA™) assay but positive by the recombinant antigen IgM test (LIAISON^®^). These samples were further tested by line blot and 83.3% (5/6) showed positive results and 16.7% (1/6) were negative. Both tests missed line blot-positive samples found by the other test, and the recombinant antigen IgM test (LIAISON^®^) came out with one probable false positive result whereas the purified flagella antigen IgM test (IDEA^®^) generated thirty probable false positive IgM results compared to the line blot.

**Table 3:** *Borrelia *specific IgM line blots. Consecutive, unselected samples.

		Line blot			
	
	Total	Positive	Equivocal	Negative	EBV
Recombinant antigen positive, Purified flagella antigen negative	6	5	-	1	-
Recombinant antigen positive, Purified flagella antigen equivocal	1	1	-	-	-
Recombinant antigen negative, Purified flagella antigen positive	33	5	3	21	4
Recombinant antigen negative, Purified flagella antigen equivocal	6	1	-	5	-

Seventeen samples were IgG negative in the purified flagella antigen test (IDEIA™) assay but IgG positive in the recombinant antigen test (LIAISON^®^), and these where further tested by line blot. Of the 17 samples 15 were available for line blot and 26.7% (4/15) were negative, 13.3% (2/15) were equivocal and 60.0% (9/15) were positive according to the definition by the manufacturer (Table 4). The recombinant antigen test (LIAISON^®^) thus seems to have a higher sensitivity for detection of *Borrelia*-specific IgG-antibodies.

**Table 4:** *Borrelia *specific IgG line blots. Consecutive, unselected samples.

		Line blot			
	
	Total	Positive	Equivocal	Negative	EBV
Recombinant antigen positive, Purified flagella antigen negative	15 out of 17	9	2	4	-
Recombinant antigen positive, Purified flagella antigen equivocal	2	2	-	-	-
Recombinant antigen equivocal Purified flagella antigen positive	1	1	-	-	-
Recombinant antigen equivocal Purified flagella antigen negative	6 out of 8	1	2	2	1

### Samples selected for IgM or IgG positivity by the purified flagella antigen assay (IDEIA™ *Borrelia burgdorferi sensu stricto*)

Five hundred and forty samples obtained from the end of January to the end of April 2004 were tested with the purified flagella antigen assay (IDEIA™) and 80 of these samples were found positive in either IgM, IgG or both and were then further tested by the recombinant antigen assay (LIAISON^®^). Fifty-three samples were IgM positive in the purified flagella antigen IgM assay (IDEIA™), but only 5.6% (3) were positive in the recombinant antigen IgM assay (LIAISON^®^) and 90.5% (48) were negative by recombinant antigen IgM assay (Table 5). In the IgG assays no sample was negative by the recombinant antigen IgG assay (LIAISON^®^) assay when positive in the purified flagella antigen IgG assay (IDEIA™). Sixty-one samples were negative in the purified flagella antigen IgG assay (IDEIA™), but were selected in this series because of IgM positivity in the initial purified flagella antigen IgM assay. Of these, 21.3% (13) were positive in the recombinant antigen IgG assay (Table 6). Of the 48 IgM positive samples 43 were available for line blot and 55.8% (24/43) were negative, 9.3% (4/43) were Epstein Barr Virus positive and potentially cross reactive, 9.3% (4/43) equivocal and 25.6% (11/43) were positive according to the definition by the manufacturer (Table 7). One sample was IgM negative in the purified flagella antigen assay but positive by the recombinant antigen IgM test and these samples showed a probable positive result in the line blot.

**Table 5:** *Borrelia *specific IgM comparing recombinant antigen assay (LIAISON^®^) and purified flagella assay (IDEIA™). Selected samples.

		Purified flagella antigen *Borrelia *IgM (IDEIA™)			
			
		POS	EQV	NEG	
Recombinant antigen *Borrelia *IgM (LIAISON^®^)	POS	3	0	1	4
	EQV	2	0	0	2
	NEG	48	16	10	74
		53	16	11	80*

*460 samples negative in the purified flagella IgM test (IDEIA™) were not tested in the recombinant antigen IgM assay (LIAISON^®^).

**Table 6:** *Borrelia *specific IgG comparing recombinant antigen assay (LIAISON^®^) and purified flagella assay (IDEIA™). Selected samples.

		Purified flagella antigen *Borrelia *IgG (IDEIA™)			
			
		POS	EQV	NEG	
Recombinant antigen *Borrelia *IgG (LIAISON^®^)	POS	15	3	13	31
	EQV	1	0	4	5
	NEG	0	0	44	44
		16	3	61	80*

*460 samples negative in the purified flagella antigen test (IDEIA™) were not tested in the recombinant antigen IgM assay (LIAISON^®^).

**Table 7:** *Borrelia *specific IgM line blots. Selected samples.

		Line blot			
	
	Total	Positive	Equivocal	Negative	EBV
Recombinant antigen positive Purified flagella antigen negative	1	1	-	-	-
Recombinant antigen equivocal Purified flagella antigen positive	2	2	-	-	-
Recombinant antigen negative, Purified flagella antigen positive	43 out of 48	11	4	24	4
Recombinant antigen negative, Purified flagella antigen equivocal	11 out of 16	7	-	4	-

Of the 13 recombinant antigen IgG positive, purified flagella antigen IgG Negative samples, 12 were available for line blot and 75% (9/12) were positive, 25% (3/12) equivocal and none were negative (Table 8).

**Table 8:** *Borrelia *specific IgG line blots. Selected samples.

		Line blot			
	
	Total	Positive	Equivocal	Negative	EBV
Recombinant antigen positive, Purified flagella antigen negative	12 out of 13	9	3	-	-
Recombinant antigen positive, Purified flagella antigen equivocal	1 out of 3	1	-	-	-
Recombinant antigen equivocal Purified flagella antigen positive	1	1	-	-	-
Recombinant antigen equivocal Purified flagella antigen negative	3 out of 4	2	-	1	-

## Discussion

The present study was performed on unselected samples submitted primarily from general practice and thus reflects the results of everyday practice in a diagnostic laboratory where most samples will have a low pretest probability of being from a patient with an acute borrelia infection. It is important to evaluate diagnostic test on unselected, population based samples to obtain information on how the test discriminate between diseased and normal groups. If assays are only evaluated on sera from clearly defined clinical cases, the true performance on samples submitted from everyday clinical practice where the diagnosis is unresolved and with a low pretest probability, will not be known [19]. It is therefore important to minimise the number of false positive IgM and IgG samples to avoid uncertainty for the physician and anxiety by the patient. If truly infected with *Borrelia*, a new sample two to four weeks later will in most cases have developed *Borrelia*-specific IgG-antibodies [15].

The flagella based assay tested showed more IgM-positive and IgG-negative results compared to the recombinant antigen based assay, and using the immunoblot (line blot) analysis as a independent reference, most of these reactions appear to be false positive.

Advantage of immunoblots (line blots) has primarily been evaluated on well defined serum collections. This introduces a selection bias because samples from unclear clinical cases are excluded [17]. Such studies will invariably show much better performance compared to results from consecutive, unselected samples with a low pretest probability. This could explain why immunoblots have shown good performance on selected samples from clinical well defined cases, but are less useful in daily practice [20].

Based on our comparison the positive outcome from the purified flagella IgM assay probably has a large proportion of false positive results using the EcoLine assay as a reference test. In addition, the recombinant antigen IgG assay has a better sensitivity compared to the purified flagella antigen test, but as a result of this also a slightly lower specificity. There are considerable variation in the *B. garinii *OspA surface antigen, and furthermore a study found substantial genetic diversity in the *OspC *gene between different isolates [21]. Other antigens utilized in diagnostic assays include the VlsE1 of *B. burgdorferi *sensu stricto and *B. garinii *[22,23].

OspC is an important antigen in the early antibody response and therefore a major target for Borrelia-specific IgM-antibodies [24-26]. False positive IgM reactions to the OspC antigen have been reported from enzyme-immunoassays [27,28] and immunoblots [29], One study found a problem of cross reactivity between IgM antibodies from *Yersinia *and *Borrelia *[30], but this result has not been confirmed in other studies.

The value of immunoblotting has been questioned by a European multicentre study, which showed considerable variability of the results between centres, and the diagnostic sensitivity and specificity has still not been determined [31]. One study compared immunoblotting reactivity against the p17 antigen of *B. afzelii *and *B. garinii *on samples from asymptomatic persons and found large differences, which could indicate unspecific false positive, reactivity [32].

However, a positive immunoblot increase the probability that the primary test was a true positive, but is not a definitive test which can be used as a golden reference standard. A recent study of Western blotting found that using a mixture of *B. burgdorferi *sensu stricto and *B. afzelii *antigens the diagnosis was improved in up to 70% of sera with borderline results from the enzyme-immunoassays using a purified flagella antigen (Zeus Scientific, Raritan, United States) [13].

## Conclusion

Our study demonstrate that compared to immunoblots, an enzyme-immunoassay based on recombinant antigens (LIAISON^®^) has fever false positive IgM results and better IgG sensitivity compared to a flagella-based assay (IDEIA™). The study showed that for IgM class antibody testing one kit is not enough because there is large antigenic variation in the OspC protein. Thus, tests using recombinant antigens may have a lower sensitivity. Contrary, sharing of antigenic epitopes on flagella among various bacterial species may result in a lack of specificity and to a smaller extent lack sensitivity of tests utilizing purified flagella antigens.

## Competing interests

Francesco Capuano is employed by DiaSorin S.p.a., Saluggia, Italy. Svend Ellermann-Eriksen has had minor consultant activities for DakoCytomation, Glostrup, Denmark. Eskild Petersen and Martin Tolstrup have no conflicts of interest. We have no stocks or shares in an organization which may in any way gain or lose financially from the publication of this manuscript. We do not hold or are currently applying for any patents relating to the content of the manuscript. We have not received reimbursements, fees, funding, or salary from an organization that holds or has applied for patents relating to the content of the manuscript. The authors have no non-financial competing interests (political, personal, religious, academic, ideological, intellectual, commercial or any other) to declare in relation to this manuscript.

## Authors' contributions

All authors contributed to the study. EP developed the protocol with SE-E and wrote the paper, FC performed the data analysis and MT performed the line-blots. All authors participated in the writing of the paper.

## Editorial Note

There is ongoing disagreement between the reviewer and authors regarding the analysis of the data presented in this study. The editors have decided to publish the article with the authors' interpretation. We encourage our readers to access the prepublication history alongside this article, so that you may form your own opinions regarding the analysis and interpretation of the data. We also encourage you to use our comments system to send us your thoughts on this matter.

## Pre-publication history

The pre-publication history for this paper can be accessed here:


